# Long Non-coding RNA RUNDC3A-AS1 Promotes Lung Metastasis of Thyroid Cancer via Targeting the miR-182-5p/ADAM9

**DOI:** 10.3389/fcell.2021.650004

**Published:** 2021-05-11

**Authors:** Dawei Ma, Yan Zhu, Xiao Zhang, Jia Zhang, Wei Chen, Xinyuan Chen, Yichun Qian, Yanbin Zhao, Tingting Hu, Zhangyu Yao, Wei Zhao, Yuan Zhang, Fangzhou Liu

**Affiliations:** ^1^Department of Pathology, The Affiliated Cancer Hospital of Nanjing Medical University, Jiangsu Cancer Hospital, Jiangsu Institute of Cancer Research, Nanjing, China; ^2^Department of Pathology, The First Affiliated Hospital of Nanjing Medical University, Nanjing, China; ^3^The Key Laboratory of Antibody Technology, National Health Commission and Nanjing Medical University, Nanjing, China; ^4^Department of Positron Emission Tomography/Computed Tomography (PET/CT) Center, The Affiliated Cancer Hospital of Nanjing Medical University, Jiangsu Cancer Hospital, Jiangsu Institute of Cancer Research, Nanjing, China; ^5^Department of Head and Neck Surgery, The Affiliated Cancer Hospital of Nanjing Medical University, Jiangsu Cancer Hospital, Jiangsu Institute of Cancer Research, Nanjing, China; ^6^School of Laboratory Medicine/Sichuan Provincial Engineering Laboratory for Prevention and Control Technology of Veterinary Drug Residue in Animal-Origin Food, Chengdu Medical College, Chengdu, China

**Keywords:** thyroid cancer, lncRNA RUNDC3A-AS1, miR-182-5p/ADAM9, *in vivo* mice model, metastasis

## Abstract

Long non-coding RNAs (lncRNAs) have been identified as influential indicators in variety of malignancies. Among which, LncRNA RUNDC3A-AS1 is reported to upregulate in thyroid cancer. However, the expression pattern and the pathological function of lncRNA RUNDC3A-AS1 in thyroid cancer is unclear. In this study, we examined the expression levels of lncRNA RUNDC3A-AS1 in the thyroid cancer tissues and cell lines via RT-qPCR analysis. The effects of RUNDC3A-AS1 on thyroid cancer cell metastasis were detected by transwell chamber assay, scratch assay *in vitro* and lung metastasis model *in vivo*. The results indicated that RUNDC3A-AS1 was highly expressed in the thyroid cancer tissues and cell lines. Functionally, knockdown of RUNDC3A-AS1 could repress the migration and invasion of thyroid cancer cells *in vitro*, and inhibit thyroid cancer metastasis to lung *in vivo*. Mechanistically, RUNDC3A-AS1 served as an inhibitor of miR-182-5p in tumor tissues and cell lines. RUNDC3A-AS1 inhibited the expression of miR-182-5p to increase the expression level of ADAM9, thus further aggravating the malignancy of thyroid cancer. Therefore, the RUNDC3A-AS1/miR-182-5p/ADAM9 axis may be a potential therapeutic target for the treatment of thyroid cancer metastasis.

## Introduction

Thyroid carcinoma (TC) is the most common endocrine malignancy and its incidence is annually increasing in the world (Yapa et al., 2017; Baloch and LiVolsi, 2018). According to pathological types, thyroid cancer can be classified into papillary thyroid cancer (PTC), follicular thyroid cancer (FTC), and anaplastic thyroid cancer (ATC). Among them, PTC accounts for 75–80% and thus is the predominant thyroid cancer (La Vecchia et al., 2015; Mao and Xing, 2016). At present, chemotherapy, surgical resection and radioactive iodine treatment are the main treatments for thyroid cancer (Applewhite et al., 2016; Cabanillas and Habra, 2016; Kim and Kim, 2016), but undesirable side effects of chemotherapy and thyroid surgery-related complications seriously affect the life quality of patients (Applewhite et al., 2016; Giuffrida et al., 2016). However, the molecular mechanism of thyroid carcinoma pathogenesis is unclear (Zaballos and Santisteban, 2017). Therefore, it is urgent to better understand the molecular mechanism of initiation and progression of thyroid cancer, which may contribute to the diagnosis and treatment of this cancer.

Over the past decades, a large number of non-coding transcripts are transcribed from human genome (Derrien et al., 2012). After annotation of these non-coding transcripts, numerous non-coding RNAs such as microRNAs, long non-coding RNAs (lncRNAs) and pseudogenes have been discovered (Harrow et al., 2012; Pei et al., 2012). Long non-coding RNAs (IncRNAs) are a group of non-coding RNAs composed of >200 nucleotides, which possess multiple biological functions, including the regulation of cell cycle and cellular differentiation (Ponting et al., 2009). Recent studies have revealed that lncRNAs exert an important role in the development and progression of various cancers (Zhang et al., 2016; Huang et al., 2018; Li et al., 2019; Qi et al., 2019). At present, some lncRNAs have been reported expression abnormally in thyroid cancer, such as lncRNA H19, lncRNA LINC00271, and lncRNA HAS2-AS1 (Luo et al., 2017; Wang et al., 2017). Accumulating studies have reported that the thyroid cancer patients with higher RUNDC3A-AS1 can decrease the survival rate (Guo et al., 2019; Lu et al., 2018). Nevertheless, to date, the functional role of this lncRNA in the metastasis of thyroid cancer is unclear.

In this study, we revealed that the key functions of RUNDC3A-AS1 on the migration and invasion of thyroid cancer *in vitro* and *in vivo*. We found that RUNDC3A-AS1 was highly expressed in thyroid cancer tissues and cell lines. Knockdown of RUNDC3A-AS1 decreased cell migration and invasion of thyroid cancer and alleviated tumor metastasis to lung through regulation of miR-182-5p/ADAM9 axis. Therefore, the main objective of the study was to decipher the roles of RUNDC3A-AS1-miR-182-5p-ADAM9 pathways in thyroid cancer, thereby providing a novel molecular mechanism correlated with the pathology of thyroid cancer and may provide a new direction for the treatment of thyroid cancer metastasis.

## Materials and Methods

### Collection of Tissue Specimens

All the thyroid cancer tissues and paired peritoneal tissues (*n* = 30) were obtained from patients (13 males and 17 females) with thyroid cancer diagnosed between May 2018 and October 2019 at the Affiliated Cancer Hospital of Nanjing Medical University and Jiangsu Cancer Hospital and Jiangsu Institute of Cancer Research. The average age of male patients were 43.6 years (range, 36–48 years) and the average age of female patients were 45.2 years (ranges, 38–51 years). The inclusion criteria were as follows: all patients didn’t receive any therapy (radiotherapy or chemotherapy) before surgery, final diagnosis confirmed by routine pathological examination and the age range 30–55 years. All the protocols for the usage of patient samples were approved by the Medical Ethics Committee of the Affiliated Cancer Hospital of Nanjing Medical University and Jiangsu Cancer Hospital and Jiangsu Institute of Cancer Research. Informed consent was signed by all patients who participated in the study. The research was conducted in accordance with the World Medical Association Declaration of Helsinki.

### Cell Culture and Treatment

Human normal thyroid cell line Nthy-ori3-1 (derived from human thyroid follicular epithelial normal cells) and human thyroid cancer cell lines (BC-PAP, K1, and TPC-1) were purchased from Chinese Academy of Sciences, Shanghai Institute of Biochemistry and Cell Biology (Shanghai, China). Cells were cultured in Roswell Park Memorial Institute (RPMI, Keygen, Nanjing, China) 1640 complete medium supplemented with 10% fetal bovine serum (FBS), 100 U/mL penicillin and 100 μg/mL streptomycin in an incubator at 37°C with 5% CO_2_ and saturated humidity. The CO_2_ cell incubator purchased from Forma Scientific UK. ABI7300 fluorescence quantitative PCR instrument was purchased from Applied Biosystems Inc.

### RNA Extraction and RT-qPCR

We used TRIZOL reagent (Thermo Fisher Scientific, United States) to extract total RNA by in cells and tissues. Taqman probes (Thermo Fisher Scientific, United States) were used to quantify miRNAs. Briefly, 1 μg of total RNA was transcribed to cDNA using AMV reverse transcriptase (Takara, Japan) and a RT primer. The reaction conditions were: 16°C for 30 min, 42°C for 30 min, and 85°C for 5 min. RT-q PCR was performed using a Taqman PCR kit on an Applied Biosystems 7300 sequence detection systems (Thermo Fisher Scientific, United States). The reactions were performed in a 96-well plate at 95°C for 10 min, followed by 40 cycles of 95°C for 10 s and 60°C for 1 min. The primer set for each gene is listed below. RUNDC3A-AS1, forward 5’-GAUCAAUACCAAUACGACA-3’, reverse 5’-UUGGAUAUCUAGUUAACUC-3’; miR-182-5p, forward 5’-ACACTCCAGCTGGGTTTGGCAATGGTAGAA CTCAC-3’, reverse 5’-CTCAACTGGTGTCGTGGA-3’; ADAM9, forward 5’-CCCCCAAATTGTGAGACTAAG-3’, reverse 5’-TCC CGTCCCTCAATGCAGTAT-3’; U6 forward 5’-CTCGCTT CGGCAGCACA-3’, reverse 5’-AACGCTTCACGAATTTGCGT-3’; GADPH forward 5’-AATCCCATCACCATCTTC-3’, reverse 5’-AGGCTGTTGTCATACTTC-3’.

U6 and GADPH were used as an internal control. PCR products were electrophoresed on 1.5% agarose gel. The fold change in gene expression was calculated using 2^–Δ^ ^Δ^ ^CT^ method after normalizing to the expression level of U6 and GADPH.

### Cell Counting Kit-8 Assay

The number of viable cells of K1 and TPC-1 was detected by the CCK-8 (Keygen, Nanjing, China). In short, the cells adjusted to the appropriate concentration were inoculated on 96-well plates and treated accordingly. Then, each well was added with CCK-8 solution and incubated for 2 h in the dark. Finally, the optical density at 450 nm was measured.

### Clone Formation Assay

The cells were seeded in 6-well plates at a density of 200 cells each well. The transfected cells were kept at 37^*o*^C in 5% CO_2_ for 14 days, and the medium was changed every 2 days. The forming colonies were fixed using 70% ethanol, followed by staining with 0.5% crystal violet. The colony consisting of 50 cells were counted using Image J software (NIH, Bethesda, MD, United States).

### Scratch Assay

When cells reached 90% confluence, a single wound was created and phase-contrast images were digitally photographed 24 and 48 h after incubation. The original opening distances of the wound were set as 100%. The opening distances after 24 or 48 h were measured from three areas randomly selected per well, and the distances in three wells of each group were quantified and normalized by the original opening distances. The experiment was performed three times in triplicate, and the percentage of the migration rate was calculated by measuring the length of cell migration and expressed as a percentage compared to the control group. Migration rates = (treatment group cell migration distance/control group migration distance) × 100%.

### Transwell Chamber Assay

An 8μm pore size transwell chamber mixed with Matrigel (Keygen, Nanjing, China) was used for transwell chamber assay. Cells were digested and counted. A total of 1 × 10^6^ cells in 100 μL medium supplemented without FBS were plated in the upper chamber and 500 μL medium supplemented with 10% FBS was covered on the bottom chambers as chemo attractant. After 24 h incubation in a humidified incubator, non-migratory cells on the upper membrane surface were carefully removed, and those on the bottom surface were fixed with 4% polyoxymethylene (Sigma, MO, United States) and stained with 0.1% crystal violet (Sigma, MO, United States) for 15 min. Cells were counted by photographing 5 random fields under a microscope (BX53, Olympus, Tokyo, Japan) at 400 × magnification and images were record.

### Western Blot Assay

The cells were lysed with RIPA kit (Beyotime, Shanghai, China). Proteins were isolated from the cell lysis buffer and quantified using the BCA protein assay kit (Beyotime, Shanghai, China). After that, Equal amount of protein (30 μg) proteins were separated by 10% SDS-PAGE gel and transferred onto a nitrocellulose membrane by means of wet transfer. Membrane blockade was conducted 5% BSA for 1 h at room temperature and incubated with primary antibodies (1:1,000, Abcam, Cambridge, MA, United Kingdom): rabbit antibodies to Cox-2 (ab15191), MMP-2 (ab97779), MMP-9 (ab38898), E-cadherin (ab40772), N-cadherin (ab18203), Snail + Slug (ab180714), ADAM9 (ab186833), and β-actin (ab8227) overnight at 4°C. The membranes were then incubated with the horseradish peroxidase (HRP)-conjugated goat anti-rabbit secondary antibody to IgG (1:5,000, Abcam, Cambridge, MA, United Kingdom). The results were visualized with an exposure machine, with β-actin regarded as an internal control. The film was scanned, the gray value was measured using the Wes automatic protein blot quantification analysis system, after which the relative ratio was calculated and subsequently compared with the internal reference. The experiment was repeated 3 times in each group.

### Dual-Luciferase Reporter Gene Assay

Bioinformatics prediction website was used to ascertain as to whether binding sites existed between RUNDC3A-AS1 and miR-182-5p as well as between miR-182-5p and 3’-untranslated region (3’-UTR) of ADAM9. Next, pmirGLO dual-luciferase miRNA target expression vector (Keygen, Nanjing, China) was performed to construct wild type-RUNDC3A-AS1 (Wt-RUNDC3A-AS1) and mutant type- RUNDC3A-AS1 (Mut-RUNDC3A-AS1) vectors. The binding site between RUNDC3A-AS1 and miR-182-5p was determined by means of dual-luciferase reporter gene assay. A full length ofRUNDC3A-AS1 gene was inserted between the two enzyme sites, *Xho*I and *Xba*I. The PCR products were detached by *Xho*I and *Xba*I and sub-cloned into thepsiCHECK-2 vector. The cells were seed into a 6-well plate with 1 × 10^6^cells per well and transfected in accordance with the aforementioned method. The successfully transfected cells were collected after a 48 h culture period. The effects ofmiR-182-5p on luciferase activity of 3’-UTR of ADAM9 was detected based on the instructions provided by the dual-luciferase detection kit (Keygen, Nanjing, China). Glomax20/20 luminometer (Yuanpinghao, Beijing, China) was utilized for fluorescence intensity determination. The experiment was repeated 3 times.

### Immunocytochemistry

After K1 cells and TPC-1 cells were treated, cells were fixed in 4%paraformaldehyde for 15 min at room temperature. The fixed cells were blocked for1 h with 5% normal goat serum in PBS and incubated with a diluted solution of the primary antibody (1:100, ab71333, Abcam, MA, United States) at 4°C overnight. Cells were then washed in PBS for 3 times and incubated for 1 h with secondary biotin-labeled goat anti-rabbit antibody to immunoglobulin G (1:1,000, ab6721, Abcam, MA, United States). Nuclei were counterstained with 4’, 6-diamidino-2-phenylindole (DAPI) (Beyotime Biotechnology, Shanghai, China). Preparations were then observed with a fluorescent microscope (Leica DM16000B, Germany) and images were record.

### Subcellular Fractionation and Localization

Nuclear and cytosolic fractions were separated using the Nuclear and Cytoplasmic Extraction Kit (Beyotime Biotechnology, Shanghai, China). Cells (1 × 10^7^) were harvested, resuspended in 1 mL of Nc-buffer A and 55 μL of Nc-buffer B, and incubated for 20 min on ice. Cells were then centrifuged for 15 min at 1,2000 r, the resulting supernatants (containing the cytoplasmic component) and nuclear pellets were used for RNA extraction. The extracted nuclear and cytoplasmic protein fractions were analyzed by RT-qPCR.

### Mouse Xenograft Model

K1 cells stably transfected with sh-lncRNA RUNDC3A-AS1, sh-NC or empty vector in PBS were injected into tail vein (1 × 10^6^ cells/mouse) of adult (6-week-old) BALB/c nude mice. Every group has 6 mice for experiment. The metastasis was observed at 8 weeks after injection. All animals were raised in the Medical Ethics Committee of the Affiliated Cancer Hospital of Nanjing Medical University & Jiangsu Cancer Hospital & Jiangsu Institute of Cancer Research. After the experiment, the mice in each group were euthanized by CO_2_ asphyxiation to obtain specimens. Half of every tumor was fixed by 4% paraformaldehyde for histomorphological analysis, and half was stored in liquid nitrogen for further study. All of the animal experiments conformed to the Guide for Care and Use of Laboratory Animals, and were approved by the Institutional Committee of Laboratory Animal Experimentation at the Medical Ethics Committee of the Affiliated Cancer Hospital of Nanjing Medical University and Jiangsu Cancer Hospital and Jiangsu Institute of Cancer Research.

### Hematoxylin Eosin (HE) Staining Assay

The lung tissues slices were stained with hematoxylin for 5 min, then rinsed for 1 min, and returned to blue by 1% ammonia (30 s). Afterward, slices were flushed with running water (1 min). Furthermore, slices were stained by 0.5% HE (for 1 min), rinsed (for 30 s), made into transparent, and finally mounted with neutral gum.

### Masson Staining Assay

Isolated lung tissues were fixed in 4% neutral formalin and embedded in paraffin. Then the sections (5 μm) were stained with Masson trichrome solutions. Images were obtained using a light microscope (BX53, Olympus, Tokyo, Japan).

### Statistical Analysis

All experiment data were analyzed using the Statistic Package for Social Science (SPSS) 19.0 statistical software (IBM Corp., Armonk, NY, United States). The experiments were repeated 3 times. Measurement data were expressed as mean ± standard deviation (SD). Comparisons between two groups were analyzed by *t*-test. Comparisons among multiple groups were analyzed by one-way analysis of variance (ANOVA), and tumor volumes at different time points were compared by repeated measurement ANOVA, followed by Tukey’s *post hoc* test. A *p* < 0.05 was considered to be statistically significant.

## Results

### The Expression Level of RUNDC3A-AS1 Is Upregulated Both in Thyroid Cancer Tissue and Its Cell Lines

**FIGURE 1:**
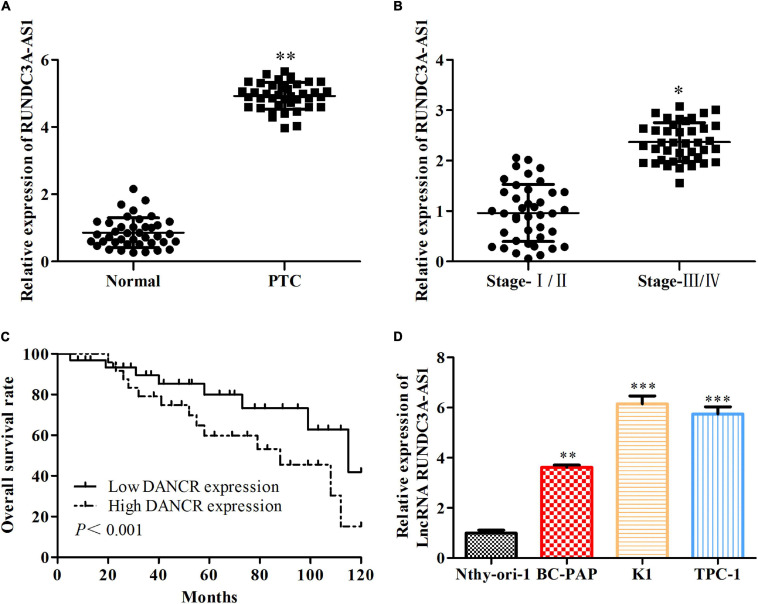
The expression level of RUNDC3A-AS1 is upregulated both in thyroid cancer tissue and its cell lines. **(A)** The expression level of lncRNA RUNDC3A-AS1 in tumor and para-carcinoma tissues. **P < 0.01 vs. the normal tissue. **(B)** The relationship between expression of lncRNA RUNDC3A-AS1 and different TNM stages of thyroid cancer.*P < 0.05, vs. the normal tissue. **(C)** The relationship between expression of lncRNA RUNDC3A-AS1 and overall survival of thyroid cancer patients in TGGA database. **(D)** The expression level of lncRNA RUNDC3A-AS1 in normal thyroid cells and thyroid cancer cell lines. **P < 0.01, ***P < 0.005 vs. the Nthy-ori3-1 cell line. The data were presented as mean ± *SD* and the experiments were repeated 3 times.

To evaluate the potential regulatory roles of RUNDC3A-AS1 in thyroid cancer, we first measured its expression pattern in the 40 pairs of thyroid cancer tissues and normal tissues. RT-qPCR analysis indicated that the expression of RUNDC3A-AS1 was increased in thyroid cancer tissues, when compared with the normal tissues (Figure 1A). Moreover, the level of RUNDC3A-AS1 was elevated with the progression of the stage of thyroid cancer (Figure 1B). Additionally, by employing the TGGA database, we further analyzed the relationship between RUNDC3A-AS1 and thyroid cancer prognosis. The results showed that patients with high RUNDC3A-AS1 expression exhibited significantly lower overall survival rate than patients with low RUNDC3A-AS1 expression (Figure 1C). At last, RT-qPCR analysis was used to examine the expression level of RUNDC3A-AS1 in human normal thyroid cell line Nthy-ori3-1 and thyroid cancer cell lines (BC-PAP, K1, and TPC-1). Compared with Nthy-ori3-1 cell line, the RUNDC3A-AS1 expression in human thyroid cancer cell lines was markedly increased. Of note, since the expression levels of RUNDC3A-AS1 were higher in K1 and TPC-1 cells (Figure 1D), we selected these two cell lines for further investigation. All these results revealed that RUNDC3A-AS1 was positively correlated with thyroid cancer progression.

### Knockdown of RUNDC3A-AS1 Represses Proliferation, Migration, and Invasion of Thyroid Cancer Cells *in vitro*

**FIGURE 2:**
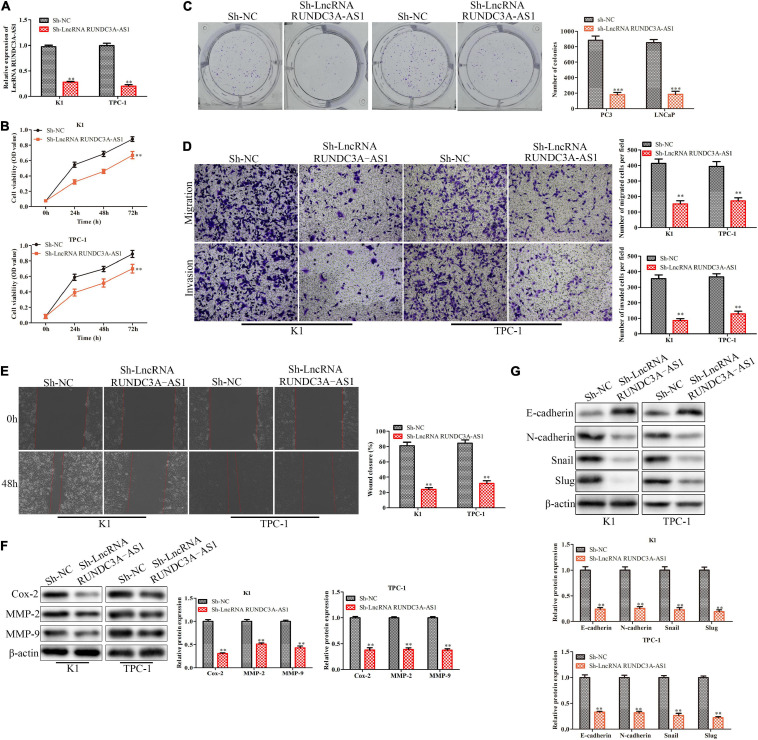
Knockdown of RUNDC3A-AS1 represses proliferation, migration and invasion of thyroid cancer cells *in vitro.* K1 and TPC-1 cells were transfected with either RUNDC3A-AS1 shRNAs or negative control (NC) shRNAs. **(A)** RT-qPCR analysis of the cell transfection. **(B)** CCK-8 assay for cell viability. **(C)** Clone formation assay for cell proliferation. **(D)** Transwell chamber assay for cell migration and invasion. **(E)** Cell capacity of migration (scratch test). **(F)** Western blot analysis of the expression levels of migration related proteins (Cox-2, MMP-2, and MMP-9). **(G)** Western blot analysis of the expression levels of epithelial mesenchymal transition (EMT) marker proteins (E-cadherin, N-cadherin, Snail, and Slug). **P < 0.01, ***P < 0.005 vs. the sh-NC group. The data were presented as mean ± *SD* and the experiments were repeated 3 times.

In order to evaluate the effect of RUNDC3A-AS1on cell proliferation, migration and invasion, the RUNDC3A-AS1 shRNA was transfected into cells (K1 and TPC-1). The RT-qPCR analysis indicated that the expression level of RUNDC3A-AS1 was significantly decreased in the sh-RUNDC3A-AS1-transfected cells (Figure 2A). The CCK-8 and clone formation assays showed that silencing ROUNDC3A-AS1 markedly inhibited the cell proliferation in K1 and TPC-1 cells (Figures 2B,C). The results of the transwell chamber and wound scratch assays revealed that RUNDC3A-AS1 deficiency inhibited cell migration and invasion both in K1 and TPC-1 cell lines (Figures 2D,E). At the molecular level, the expression levels of Cox-2, MMP-2, and MMP-9 proteins, which were correlated with cell migration and invasion were markedly down-regulated when RUNDC3A-AS1 was knocked down (Figure 2F). In order to verify the function of silencing RUNDC3A-AS1 in cell metastasis, we detected the epithelial-mesenchymal transition (EMT) marker protein molecule epithelial-cadherin (E-cadherin, E-cad), neural cadherin (N-cadherin, N-cad), zinc finger transcription factor Snail and zinc finger transcription factor Snail2 (Slug) expression levels in K1 and TPC-1 cells. The results indicated that the E-cadherin expression was increased while the expression levels of N-cadherin, Snail and Slug were decreased in K1and TPC-1 cells when RUNDC3A-AS1 was knocked down (Figure 2G). Hence, all the above results demonstrated that RUNDC3A-AS1 knockdown could repress migration and invasion of thyroid cancer cells (K1 and TPC-1), which indicated that RUNDC3A-AS1 was a potential essential factor for the migration and invasion of thyroid cancer cells.

### RUNDC3A-AS1 Directly Binds to miR-182-5p and Downregulates the Expression of miR-182-5p

**FIGURE 3:**
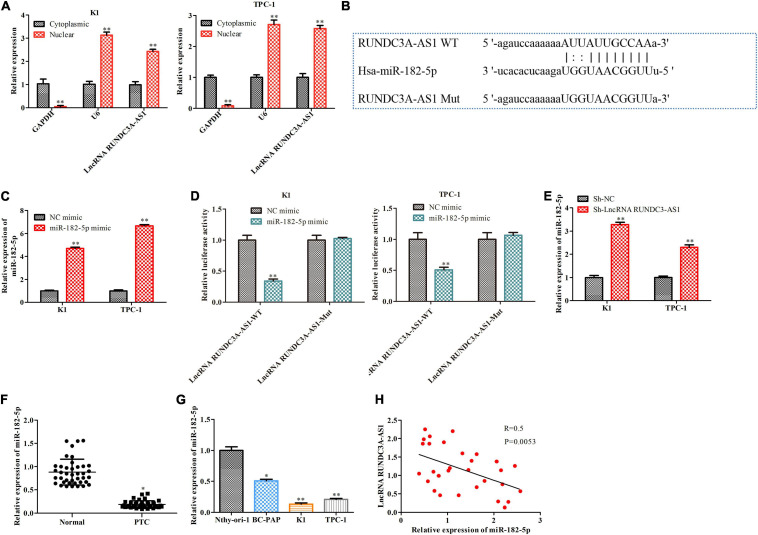
RUNDC3A-AS1 directly binds to miR-182-5p and downregulates the expression of miR-182-5p. **(A)** The expression level of RUNDC3A-AS1 in nucleus and cytoplasm was analyzed by RT-qPCR. **P < 0.01 vs. the Cytoplasmic group. **(B)** The predicted RUNDC3A-AS1 binding sites in the region of miR-182-5p and the corresponding mutant sequence were shown. **P < 0.01 vs. the Cytoplasmic group. **(C)** The relationship level of miR-182-5p both in K1 and TPC-1 cells after transfected with NC mimic and miR-182-5p mimic, respectively. **(D)** Relative values of luciferase signal. *P < 0.05, **P < 0.01 vs. the NC mimic group. **(E)** The expression level of miR-182-5p both in K1 and TPC-1 cells after transfected with sh-NC and RUNDC3A-AS1 shRNAs, respectively. **P < 0.01 vs. the sh-NC group. **(F)** The expression level of miR-182-5p in tumor and para-carcinoma tissues. *P < 0.05 vs. the normal group. **(G)** The expression level of miR-182-5p in normal thyroid cells and thyroid cancer cell lines. *P < 0.05, **P < 0.01 vs. the Nthy-ori3-1 cell line. **(H)** A negative correction between lncRNA RUNDC3A-AS1 and miR-182-5p expression. The data were presented as mean ± *SD* and the experiments were repeated 3 times.

LncRNAs have been demonstrated to serve as competing endogenous RNAs (ceRNAs), which sponge miRNAs to regulate the expression of miRNAs. Firstly, we confirmed that RUNDC3A-AS1 was mainly expressed in the cytoplasm by RT-qPCR through nuclear-plasma separation experiment (Figure 3A). Moreover, to find out the specific miRNA that was regulated by lncRNA RUNDC3A-AS1, we performed bioinformatics analysis^1^ and dual-luciferase reporter gene assay. The results of bioinformatics analysis suggested that a promising binding site existed between RUNDC3A-AS1 and miR-182-5p (Figure 3B). A recently study reported the overexpression of miR-182-5p in papillary thyroid carcinoma compared to the levels in adjacent normal tissues (Liu et al., 2017). Thus, we first validated that the miR-182-5p expression was significantly increased in the K1 and TPC-1 cells after treating miR-182-5p mimic compared to the NC mimic group (Figure 3C). Furthermore, we investigated whether RUNDC3A-AS1 could directly regulate miR-182-5p expression in the K1 and TPC-1 cells by using luciferase reporter assay. The results revealed that the relative luciferase activity of RUNDC3A-AS1-Wt was obviously decreased by miR-182-5p mimic, whereas no significant reduction was observed in the luciferase activity of RUNDC3A-AS1-Mut (Figure 3D). Additionally, we assessed the level of miR-182-5p in thyroid cancer cells (K1 and TPC-1 cells) transfected with sh-RUNDC3A-AS1. We found that the level of miR-182-5p was increased in response to RUNDC3A-AS1 knockdown (Figure 3E). Meanwhile, we also verified miR-182-5p expression in thyroid cancer tissues and thyroid cancer cell lines. RT-qPCR analysis indicated that the expression of miR-182-5p was decreased in thyroid cancer tissues and thyroid cancer cell lines (Figures 3F,G). In addition, the expression level of RUNDC3A-AS1 in thyroid cancer tissues and thyroid cancer cell lines was negatively correlated with miR-182-5p expression (Figure 3H). Taken together, these data suggested RUNDC3A-AS1 directly bound to miR-182-5p and downregulaed the expression of miR-182-5p.

### Overexpression of miR-182-5p Suppresses Migration and Invasion of Thyroid Cancer Cells *in vitro*

**FIGURE 4:**
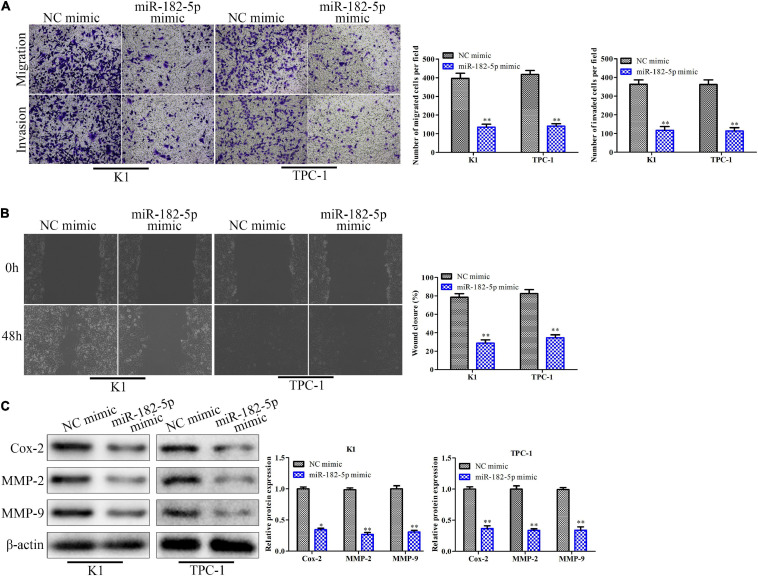
Overexpression of miR-182-5p suppresses migration and invasion of thyroid cancer cells *in vitro*. K1 and TPC-1 cells were transfected with either miR-182-5p mimic or NC mimic. **(A)** Transwell chamber assay for cell migration and invasion. **(B)** Cell capacity of migration (scratch test). **(C)** Western blot analysis of the expression level of migration related proteins (Cox-2, MMP-2, and MMP-9). *P < 0.05, **P < 0.01 vs. the NC mimic group. The data were presented as mean ± *SD* and the experiments were repeated 3 times.

To investigate the function of miR-182-5p on cell migration and invasion, the miR-182-5p mimic was transfected into K1 and TPC-1 cells and transwell chamber assays and wound scratch were performed. The results of transwell chamber assay indicated that the number of migratory cells and invasive cells in the miR-182-5p mimic-treated group was markedly reduced compared with the NC mimic group (Figure 4A). Consistently, the wound scratch assay showed that the wound closure of the distance in the miR-182-5p mimic group was significantly decreased compared to that in the NC mimic group (Figure 4B). At the molecular level, the expression levels of Cox-2, MMP-2, and MMP-9 proteins were markedly downregulated when miR-182-5p was overexpressed (Figure 4C). Collectively, overexpression of miR-182-5p could inhibit cell migration and invasion both in the K1 and TPC-1 cell lines.

### MiR-182-5p Targets ADAM9 and Causes Post-transcriptional Suppression

**FIGURE 5:**
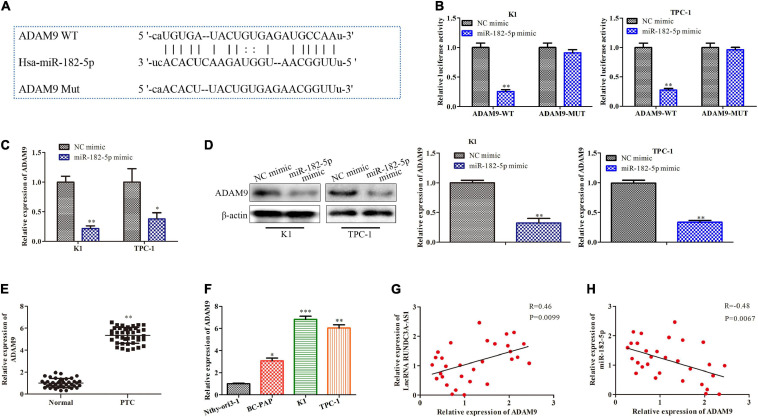
MiR-182-5p targets ADAM9 and causes posttranscriptional suppression. **(A)** The predicted miR-182-5p binding sites in the region of ADAM9 and the corresponding mutant sequence were shown. **(B)** Relative values of luciferase signal. **(C)** The expression level of ADAM9 both in K1 and TPC-1 cells after transfected with NC mimic and miR-182-5p mimic, respectively. **(D)** Western blot analysis of the expression level of ADAM9 protein. *P < 0.05, **P < 0.01 vs. the NC mimic group. **(E)** The expression level of ADAM9 in tumor and para-carcinoma tissues. **P < 0.01 vs. the normal group. **(F)** The expression level of ADAM9 in normal thyroid cells and thyroid cancer cell lines. **P < 0.01, ***P < 0.005 vs. the Nthy-ori3-1 cell line. **(G)** A positive correction between lncRNA RUNDC3A-AS1 and ADAM9 expression. **(H)** A negative correction between miR-182-5p and ADAM9 expression. The data were presented as mean ± *SD* and the experiments were repeated 3 times.

In order to explore the potential the molecular mechanism of miR-182-5p, bioinformatics analysis^1^ was used to predict potential target of miR-182-5p. ADAM9 was considered to be a potential target of miR-182-5p in thyroid cancer because it played an important role in tumor migration, invasion and metastasis (Xiong et al., 2015). As shown in Figure 5A, the 3’-UTR of the ADAM9 contained a putative binding site of miR-182-5p. The regulatory effect of miR-182-5p and ADAM9 was further validated by the dual-luciferase reporter gene assay. The results showed that miR-182-5p mimic was able to inhibit the luciferase activity of ADAM9-Wt compared with mimic-NC. However, no significance changes were observed in the luciferase activity of ADAM9-Mut, indicating that ADAM9 was a direct target of miR-182-5p in the K1 and TPC-1 cells (Figure 5B). Then, we detected the expression of ADAM9 in the K1 and TPC-1 cells through RT-qPCR and western blot. The results revealed that the expression level of ADAM9 markedly decreased in cells transfected with miR-182-5p mimic (Figures 5C,D). In addition, we validated the mRNA expression levels of ADAM9 in thyroid cancer tissues and thyroid cancer cell lines. RT-qPCR analysis indicated that the expression level of ADAM9 was markedly increased in thyroid cancer tissues and thyroid cancer cell lines (Figures 5E,F). Last but not the least, we analyzed the relationship among the expression levels of RUNDC3A-AS1, ADAM9 and miR-182-5p. The results suggested that the expression levels of RUNDC3A-AS1 in thyroid cancer tissues and cell lines were positively correlated with ADAM9 expression (Figure 5G), while the expression levels of miR-182-5p were negatively correlated with ADAM9 expression (Figure 5H). Therefore, ADAM9 was a target gene of miR-182-5p and was negatively regulated by miR-182-5p, while was positively regulated by RUNDC3A-AS1.

### LncRNA RUNDC3A-AS1 Promotes Thyroid Cancer Cell Migration and Invasion by Regulating miR-182-5p/ADAM9 Axis

**FIGURE 6:**
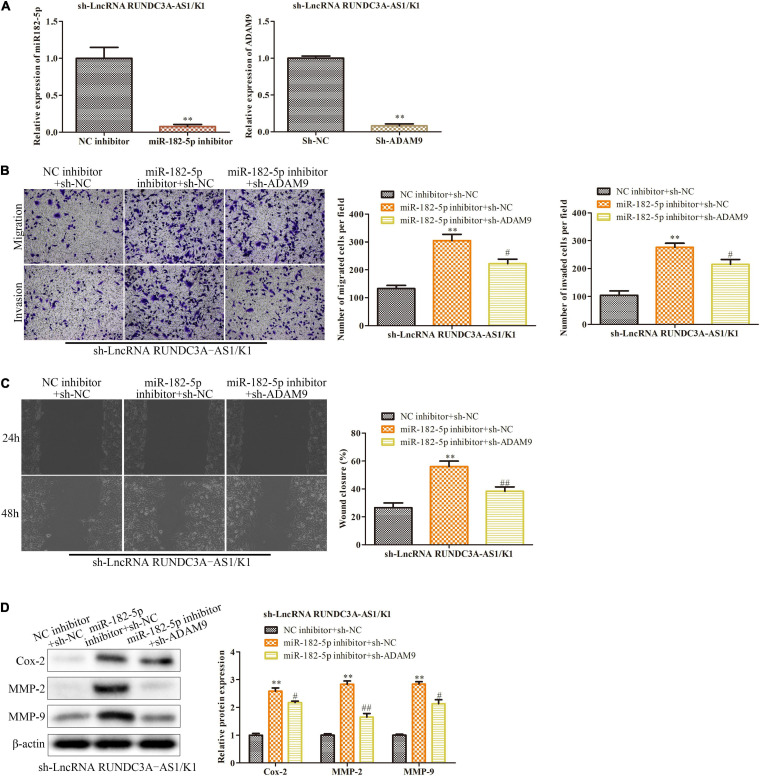
LncRNA RUNDC3A-AS1 promotes thyroid cancer cell migration and invasion by regulating miR-182-5p/ADAM9 axis. Either miR-182-5p inhibitor or ADAM9 shRNAs were transfected into the RUNDC3A-AS1 knockdown K1 cells. **(A)** RT-qPCR was performed to evaluate the efficiency of miR-182-5p inhibitor and ADAM9 knockdown. **P < 0.01 vs. the NC inhibitor group and the sh-NC group, respectively. **(B)** Transwell chamber assay for cell migration and invasion. **(C)** Cell capacity of migration (scratch test). **(D)** Western blot analysis of the expression level of migration related proteins (Cox-2, MMP-2, and MMP-9). **P < 0.01 vs. the sh-NC + NC inhibitor group. ^#^*P* < 0.05, ^##^*P* < 0.01 vs. the miR-182-5p inhibitor + sh-NC group. The data were presented as mean ± *SD* and the experiments were repeated 3 times.

To test whether miR-182-5p/ADAM9 axis was involved in RUNDC3A-AS1 promoted thyroid cancer progression, miR-182-5p inhibitors and ADAM9 shRNAs were transfected into K1 cells in the presence with RUNDC3A-AS1 shRNAs. The efficiency of miR-182-5p inhibition and ADAM9 knockdown in K1 were present in Figure 6A. The expression of miR-182-5p and ADAM9 was reduced compared to NC inhibitor group and sh-NC group, respectively. The transwell chamber and wound scratch assays indicated that miR-182-5p inhibitor could improve the ability of the migration and invasion in the K1 cells with RUNDC3A-AS1 knockdown, while these effects of RUNDC3A-AS1on the migration and invasion were partially antagonized by knockdown of ADAM9 (Figures 6B,C). At the molecular level, the expression levels of Cox-2, MMP-2 and MMP-9 proteins were markedly upregulated when miR-182-5p was inhibited and ADAM9 was knocked down (Figure 6D). Taken together, RUNDC3A-AS1 regulates thyroid cancer cell migration and invasion through miR-182-5p/ADAM9 axis.

### Knockdown of RUNDC3A-AS1 Inhibits Thyroid Tumor Metastasis to Lung *in vivo*

**FIGURE 7:**
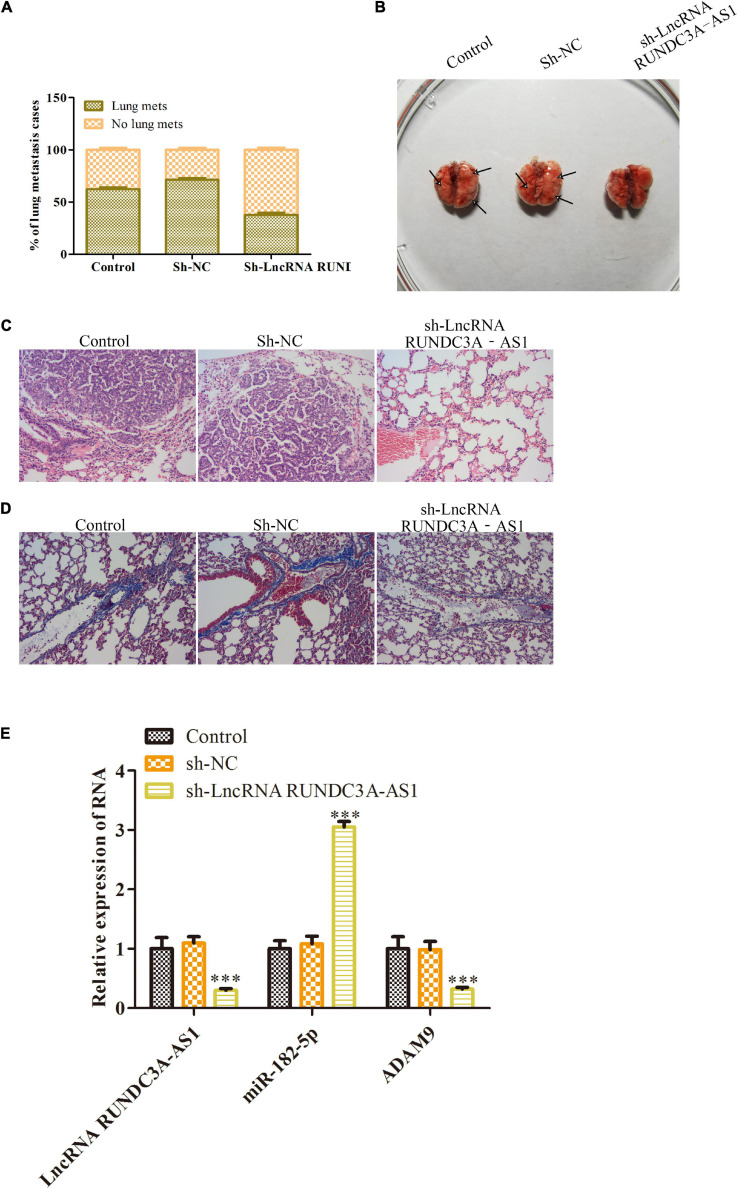
Knockdown of RUNDC3A-AS1 inhibits thyroid tumor metastasis to lung *in vivo*. K1 cells transfected with sh-RUNDC3A-AS1 and sh-NC were injected into athymic nude mice via the tail vein. **(A)** The incidence of lung metastasis in K1 thyroid tumor. **(B)** The images of lung metastasis of sh-NC and sh-RUNDC3A-AS1 groups by stereo fluorescence microscope and lung metastasis analysis of sh-NC and sh-RUNDC3A-AS1 groups. **(C)** Hematoxylin eosin (HE) staining assay. **(D)** Masson staining assay. **(E)** RT-qPCR detected the expression of RUNDC3A-AS1, miR-182-5p, and ADAM9 in indicated groups. ***P < 0.001 vs. the sh-NC group.

Given the inhibitory effects of lncRNA RUNDC3A-AS1 on cell migration and invasion, we next wanted to evaluate the effect of lncRNA RUNDC3A-AS1 on thyroid tumor metastasis *in vivo*. K1 cells transfected with sh-RUNDC3A-AS1 and sh-NC were injected into athymic nude mice via the tail vein, and the mice were imaged every week. We found that the sh-RUNDC3A-AS1 decreased the lung metastatic incidence of the K1 thyroid tumor (Figure 7A). Moreover, the mice were killed for lung metastatic analyses at 8 weeks of age. The nude mice of knockdown of RUNDC3A-AS1 were demonstrated significantly decreased lung metastatic modules compared with that of empty vector group and sh-NC group (Figure 7B). In addition, to further determine the inhibitory role of sh-RUNDC3A-AS1 in lung metastasis, HE staining and Masson staining analyses were used to observe the histopathological changes in lung tissue. As displayed in Figure 7C, knockdown of RUNDC3A-AS1 could markedly decrease the degree of lung cancer compared to the control group and sh-NC group. Furthermore, masson staining analysis revealed that the degree of pulmonary fibrosis of the knockdown of RUNDC3A-AS1 group was decreased significantly compared with that in control group and sh-NC group (Figure 7D). RT-qPCR detected the expression of RUNDC3A-AS1, miR-182-5p and ADAM9 in metastatic tumors, Figure 7E shown that the miR-182-5p expression was upregulated and ADAM9 was downregulated when RUNDC3A was blocked *in vivo*. In conclusion, lncRNA RUNDC3A-AS1 could promote thyroid tumor metastasis to lung *in vivo*.

## Discussion

Previous studies have shown that thyroid cancer is the most common endocrine malignancy in the world (Yapa et al., 2017; Baloch and LiVolsi, 2018). Over the past few decades, studies have revealed that multiple lncRNAs are abnormally expressed in thyroid cancer (Luo et al., 2017; Wang et al., 2017; Lu et al., 2018; Guo et al., 2019). Therefore, dissecting the role of lncRNAs in thyroid cancer progression is important for the identification of thyroid cancer clinical treatment. In this study, we found that the expression level of lncRNA RUNDC3A-AS1 was upregulated both in thyroid cancer tissue and cell lines. Knockdown of lncRNA RUNDC3A-AS1 could repress the migration and invasion of thyroid cancer cells *in vitro* and inhibit lung metastasis of thyroid cancer *in vivo*.

Studies have shown that miRNA have become inhibitory or carcinogenic in tumorigenesis and the expression of lncRNAs can regulate the activities of miRNAs (Jiang et al., 2015; Li et al., 2017). It is now increasingly acknowledged that lncRNAs regulate development and progression of thyroid cancer via sponging an array of downstream miRNAs (Luo et al., 2017; Wang et al., 2017). Therefore, taking in-depth study of these miRNAs provides new opportunities for developing effective techniques to prevent and treat the thyroid cancer. The downstream miRNA-182-5p in our study was significantly down-regulated in many tumors, such as hepatocellular carcinoma (Cao et al., 2018), non-small-cell lung cancer (Yang et al., 2019), renal cancer (Wang et al., 2020), gastric cancer (Sun et al., 2018), and bladder cancer (Wang et al., 2019). In this study, we found that miR-182-5p was a target miRNA of RUNDC3A-AS1. Accordingly, the expression level of miR-182-5p was down-regulated and negatively correlated with the RUNDC3A-AS1 in thyroid cancer tissues and cell lines. Luciferase assays revealed that miR-182-5p could bind to RUNDC3A-AS1 and decrease its luciferase activity in the K1 and TPC-1 cell lines, thus antagonized the effect of RUNDC3A-AS1 on the thyroid cancer cell progression. Meanwhile, overexpression of miR-182-5p could inhibit cell migration and invasion both in K1 and TPC-1 cell lines. These results indicated that RUNDC3A-AS1 could directly bind to miR-182-5p and downregulate the expression of miR-182-5p to promote thyroid cancer cell migration and invasion.

On the other hand, miR-182-5p has been proved to be a tumor suppressor, which is related with tumor cell proliferation, migration, invasion, and apoptosis (Cao et al., 2018; Sun et al., 2018; Wang et al., 2019, 2020; Yang et al., 2019). Therefore, screening out the gene target of miR-182-5p in the thyroid cancer is of great interest. In this study, given that ADAM9 is a famous oncogene in various tumors (Chiu et al., 2017; Hua et al., 2018; Liu et al., 2018; Guo et al., 2019), we demonstrated the ADAM9 was a target gene of miR-182-5p by bioinformatics and luciferase activity analyses. At the same time, the expression of ADAM9 was negatively regulated by miR-182-5p and positively regulated by RUNDC3A-AS1. More importantly, knockdown of ADAM9 reversed the neutralization effect of miR-182-5p on the RUNDC3A-AS1 knockdown-induced inhibition of thyroid cancer cell migration and invasion. Therefore, RUNDC3A-AS1 might enhance ADAM9 expression by sequestering the miR-182-5p in thyroid cancer.

In the present study, we demonstrated that the expression level of RUNDC3A-AS1 was upregulated in thyroid cancer tissues and cell lines. Moreover, knockdown of RUNDC3A-AS1 could inhibit thyroid cancer cell migration and invasion *in vitro* and repress lung metastasis of thyroid cancer *in vivo*. In addition, we found that miR-182-5p was down-regulated in thyroid cancer tissues and cell lines, and overexpression of miR-182-5p could repress thyroid cancer cell migration and invasion. Mechanistically, the effect of RUNDC3A-AS1 on the thyroid cancer was partially mediated by miR-182-5p/ADAM9 axis. Therefore, the RUNDC3A-AS1/miR-182-5p/ADAM9 axis may serve as novel biomarkers or potential targets for the treatment of thyroid cancer metastasis.

## Data Availability Statement

The raw data supporting the conclusions of this article will be made available by the authors, without undue reservation.

## Ethics Statement

The studies involving human participants were reviewed and approved by the Medical Ethics Committee of the Affiliated Cancer Hospital of Nanjing Medical University and Jiangsu Cancer Hospital and Jiangsu Institute of Cancer Research. The patients/participants provided their written informed consent to participate in this study. The animal study was reviewed and approved by the Institutional Committee of Laboratory Animal Experimentation at the Medical Ethics Committee of the Affiliated Cancer Hospital of Nanjing Medical University and Jiangsu Cancer Hospital and Jiangsu Institute of Cancer Research.

## Author Contributions

JZ and FL conceived and designed the study. XZ, WC, XC, and YQ performed the literature search and data extraction. YZha, TH, and WZ performed the literature search and data extraction. DM and YZhu drafted the manuscript. All authors read and approved the final manuscript.

## Conflict of Interest

The authors declare that the research was conducted in the absence of any commercial or financial relationships that could be construed as a potential conflict of interest.

## Footnotes

1 http://starbase.sysu.edu.cn
